# Overweight and obesity as protective factors against mortality in nonischemic cardiomyopathy patients with an implantable cardioverter defibrillator

**DOI:** 10.1002/clc.23458

**Published:** 2020-09-16

**Authors:** Bin Zhou, Shuang Zhao, Min Tang, Keping Chen, Wei Hua, Yangang Su, Silin Chen, Zhaoguang Liang, Wei Xu, Xiaoyao Li, Xiaodi Xue, Xuerong Sun, Shu Zhang

**Affiliations:** ^1^ Arrhythmia Centre, Fuwai Hospital, National Centre for Cardiovascular Diseases, State Key Laboratory of Cardiovascular Disease Chinese Academy of Medical Sciences and Peking Union Medical College Beijing China; ^2^ Department of Cardiology Shanghai Institute of Cardiovascular Diseases, Zhongshan Hospital, Fudan University Shanghai China; ^3^ Department of Cardiology Guangdong Cardiovascular Institute, Guangdong General Hospital Guangzhou China; ^4^ Department of Cardiology First Affiliated Hospital of Harbin Medical University Harbin China; ^5^ Department of Cardiology Nanjing Drum Tower Hospital Nanjing China

**Keywords:** body mass index, implantable cardioverter defibrillator, mortality, nonischemic cardiomyopathy

## Abstract

**Background:**

Previous studies have reported inconsistent results on the relationship between body mass index (BMI) and clinical outcomes in implantable cardioverter defibrillator (ICD) patients. Additionally, research on ICD patients with nonischemic cardiomyopathy (NICM) is lacking.

**Hypothesis:**

This study aimed to investigate the impact of BMI on mortality and ventricular arrhythmias (VAs) in NICM patients with an ICD.

**Methods:**

This study retrospectively analyzed the data from the Study of Home Monitoring System Safety and Efficacy in Cardiac Implantable Electronic Device‐implanted patients (SUMMIT) in China. Four hundred and eighty NICM patients with an ICD having BMI data were enrolled. Patients were divided into two groups: underweight and normal range group (BMI < 24 kg/m^2^), overweight and obese group (BMI≥24 kg/m^2^). The primary endpoint was all‐cause mortality. The secondary endpoint was the first occurrence of VAs requiring appropriate ICD therapy or shock.

**Results:**

During a median follow‐up of 61 (1‐95) months, 70 patients (14.6%) died, 173 patients (36%) experienced VAs requiring appropriate ICD therapy, and 112 patients (23.3%) were treated with ICD shock. Multivariate Cox regression modeling indicated a decreased mortality risk in the overweight and obese group compared with the underweight and normal range group (hazard ratio = 0.44, 95% confidence interval 0.26‐0.77, *P* = .003). However, the risk of VAs was similar in both groups in univariate and multivariate Cox models.

**Conclusions:**

Compared with underweight and normal weight, overweight and obesity are protective against mortality but have only a neutral impact on VAs risk in NICM patients with an ICD.

## 1 INTRODUCTION

Overweight and obesity are global health concerns, leading to an increase in morbidity and mortality.^1^ Although obesity is associated with diabetes, hyperlipidemia, and hypertension, which are harmful to cardiovascular health, a phenomenon called the “obesity paradox” has been observed in overweight and obese patients with hypertension, coronary heart disease, atrial fibrillation (AF), and heart failure.^2^, ^3^, ^4^, ^5^, ^6^, ^7^, ^8^, ^9^, ^10^, ^11^ The 'obesity paradox' occurs when a higher body mass index (BMI) leads to better clinical outcomes. For patients with an implantable cardioverter defibrillator (ICD) who lived with a potentially fatal arrhythmia or a high risk of sudden cardiac death (SCD) due to underlying heart disease, whether the “obesity paradox” exists is controversial because the relevant research results are inconsistent.^12^, ^13^, ^14^, ^15^, ^16^, ^17^ Additionally, no research has focused on ICD patients with nonischemic cardiomyopathy (NICM). Our study intended to explore the impact of BMI on clinical outcomes in NICM patients with an ICD.

## 2 METHODS

### 2.1 Study population

We conducted a retrospective analysis of data from the Study of Home Monitoring System Safety and Efficacy in Cardiac Implantable Electronic Device‐implanted patients (SUMMIT) registry in China. SUMMIT was a prospective, observational, multicenter registry intending to evaluate the safety and efficacy of a cardiac implantable electronic device with a home monitoring (HM) system in China.

Four hundred and eighty NICM patients who underwent an ICD with an HM system (Biotronik, Berlin, Germany) between May 2010 and May 2015 from the SUMMIT registry were enrolled. Cardiac resynchronization therapy defibrillator and subcutaneous implantable cardioverter defibrillator were not included. ICD implantation indications of secondary prevention and primary prevention were consistent with the ACC/AHA/HRS/ESC Guidelines.^18^, ^19^ Secondary prevention refers to prevention of SCD in those patients who have survived a prior sudden cardiac arrest or sustained ventricular tachycardia (VT) or ventricular fibrillation (VF); primary prevention of SCD refers to the use of ICDs in individuals who are at risk for but have not yet had an episode of sustained VT, VF, or resuscitated cardiac arrest.^18^ Myocardial dysfunction means myocardium associated with mechanical and/or electrical dysfunction that usually (but not invariably) exhibit inappropriate ventricular hypertrophy or dilatation and are due to a variety of causes; within this broad definition, cardiomyopathies (CM) usually are associated with failure of myocardial performance, which may be mechanical (eg, diastolic or systolic dysfunction) or a primary electrical disease prone to life‐threatening arrhythmias.^20^ NICM was defined as myocardial dysfunction in the absence of a history of significant obstructive coronary artery disease (CAD) including known chronic angina pectoris, previous myocardial infarction or unstable angina, coronary artery bypass grafting, or previous percutaneous coronary intervention with or without stenting.^21^ Patients could be included even if they were diagnosed with intermediate CAD which was not considered to be sufficient to account for the myocardial dysfunction meeting the ICD implantation criteria.^22^ BMI information was available prior to ICD implantation. BMI was calculated as weight (kg) divided by the square of the patient's height (m^2^) and shown as kg/m^2^. According to Chinese obesity working group definitions, we classified patients as underweight (<18.5 kg/m^2^), normal weight (18.5‐<24 kg/m^2^), overweight (24‐28 kg/m^2^), and obese (≥28 kg/m^2^) based on BMI.^23^ Other baseline clinical characteristics were acquired from the patients' medical records before ICD implantation. The study protocols were approved by the local hospital ethics committees and were in accordance with the Declaration of Helsinki. All patients signed informed consent forms before the study.

### 2.2 Device settings

Programming settings were as follows: the basic pacing rate was 40 to 60 beats per minute (bpm), target VT monitor zone was 140 to 170 bpm, target VT therapy zone was over 170 to 210 bpm, and VF zone was over 210 bpm. In VT therapy zone, 2 to 3 bursts of anti‐tachycardia pacing (ATP) were delivered, followed by high‐energy shock for persisting episodes. In VF zone, high‐energy shock alone was used. The detection interval was 26 beats in VT zone with a 20 beats redetection. And the detection interval was 12 out of 16 beats in VF zone. Other programmable parameters are determined by individual doctors. VT refers to spontaneous ventricular depolarization with a frequency of more than 100 bpm, for 3 or more consecutive times. The duration of QRS is usually wider than 120 ms. VF refers to the disordered agitation of the ventricle, which leads to the regular and orderly agitation and the disappearance of the systolic and diastolic function of the ventricle. Its electrocardiogram (ECG) is as follows: a constant shift in axis and morphology of the electrogram is accompanied by marked and variable changes in electrogram amplitude.

In our study, ICD was equipped with Biotronik SMART algorithm which could automatically analyze the waveform and frequency of ECG to distinguish VT/VF and supraventricular tachycardia (including atrial fibrillation, atrial flutter, and sinus tachycardia). Additionally, the tachycardia events could be monitored by the ICD and automatically transmitted to the home monitoring system. Two cardiologists reviewed the intracardiac electrograms (IEGM) of tachycardia events in a blinded manner to further confirm the event as VT/VF or supraventricular tachycardia and analyzed the VAs to assess whether the patient received the appropriate ICD therapy by ATP or shock. When there was a disagreement on the IEGM reading and ICD therapy, a third cardiologist was responsible for a conclusive opinion.

### 2.3 End points and follow‐up

The primary endpoint was all‐cause death. The secondary endpoints were: (a) the first occurrence of VAs requiring appropriate ICD therapy by ATP or shock and (b) the first occurrence of VAs requiring appropriate ICD shock. VAs with a heart rate slower than 140 bpm and nonsustained VT with an episode of VT satisfying the ICD programmed detection criteria but self‐terminated before the delivery of ICD therapy were not included in our analysis. Inappropriate device therapies were also evaluated in the same protocol and inappropriate events detections were excluded. The interval from ICD implantation to the first occurrence of VAs requiring appropriate ICD therapy or ICD shock was recorded in the HM system. Routine follow‐ups were conducted, and patient status was confirmed via phone calls in the events their transmissions were disrupted. We contacted the family members or witnesses to carefully evaluate the date of death once a patient died.

### 2.4 Statistical analysis

Continuous variables were summarized as the mean ± SD, and discrete variables were summarized as frequency and percentage. One‐way analysis of variance with Bonferroni post hoc testing was used to test for differences in continuous variables, and *χ* ^2^ or Fisher's exact test was used for categorical variables. Kaplan‐Meier curves for cumulative incidence of all‐cause death and VAs over time were plotted (log‐rank test used). Cox proportional hazard models were conducted for all‐cause death and VAs. All variables that had a statistically significant effect at the 0.05 level in the univariate Cox model were introduced into a multivariate Cox model (forced‐entry method). BMI ≥24 kg/m^2^ was forced into the multivariate Cox model due to the analysis's primary interest was the association of BMI ≥24 kg/m^2^ with the clinical outcome. A *P* value <.05 from a two‐sided test was considered to indicate statistical significance. All statistical analyses were performed with SPSS Statistics version 23.0 (IBM Corp., Armonk, NY) and by the GraphPad Prism software version 8.0 (GraphPad Software, La Jolla, CA).

## 3 RESULTS

### 3.1 Patient demographics

Four hundred and eighty NICM patients with an ICD were enrolled. On the basis of BMI, we classified the patients as underweight (25,5.2%), normal range (260,54.2%), overweight (174, 36.3%), and obese (21, 4.4%). Due to the low proportion of patients in the underweight and obese groups, we divided the overall patient population into two groups: underweight and normal group, overweight and obese group. There were no significant differences in baseline characteristics between the two groups (Table 1).

**TABLE 1:** Baseline characteristics of the study population according to BMI

	Total population (n = 480)	Underweight and normal (BMI < 24 kg/m^2^) (n = 285)	Overweight and obese (BMI≥24 kg/m^2^) (n = 195)	*P* value
Demographics				
Age at implantation, years	57.4 ± 14.7	57.0 ± 15.5	57.9 ± 13.4	.538
Male (n, %)	326(67.9)	187(65.6)	139(71.3)	.191
NYHA, classes III‐IV (n, %)	158(32.9)	97(34)	61(31.3)	.528
Primary prevention (n, %)	206(42.9)	123(43.5)	83(42.6)	.845
SBP, mmHg	123.8 ± 16.6	122.9 ± 16.3	125.2 ± 17.0	.129
DBP, mmHg	76.6 ± 10.5	76.1 ± 10.7	77.3 ± 10.2	.211
QRS duration, ms	110.6 ± 27.4	109.2 ± 24.8	112.8 ± 31.0	.292
Comorbidities (n, %)				
Hypertension	100(20.8)	59(20.7)	41(21.0)	.932
Diabetes	26(5.4)	16(5.6)	10(5.1)	.817
Atrial fibrillation	39(8.1)	21(7.4)	18(9.2)	.463
Stroke	5(1)	3(0.4)	4(2.1)	.164
Preimplant syncope	113(23.5)	69(24.2)	44(22.6)	.676
Echocardiography				
LVEF, %	48.4 ± 15.0	47.9 ± 15.1	49.0 ± 14.8	.424
LVEDD, mm	54.0 ± 11.8	53.3 ± 12.0	55.0 ± 11.5	.132
Medications (n, %)				
β‐Blocker	261(54.4)	156(54.7)	105(53.8)	.847
Amiodarone	143(29.8)	88(30.9)	55(28.2)	.530
ACEI or ARB	126(26.3)	67(23.6)	59(30.3)	.104
Loop diuretic	85(17.7)	49(17.2)	36(18.6)	.701
Spironolactone	110(22.9)	65(22.8)	45(23.1)	.945

Abbreviations: ACEI, angiotensin‐converting enzyme inhibitor; ARB, angiotensin receptor blocker; BMI, body mass index; LVEF, left ventricular ejection fraction; LVEDD, left ventricular end‐systolic dimension; NYHA, New York Heart Association.

### 3.2 Influence of BMI on all‐cause death and VAs

**FIGURE 1:**
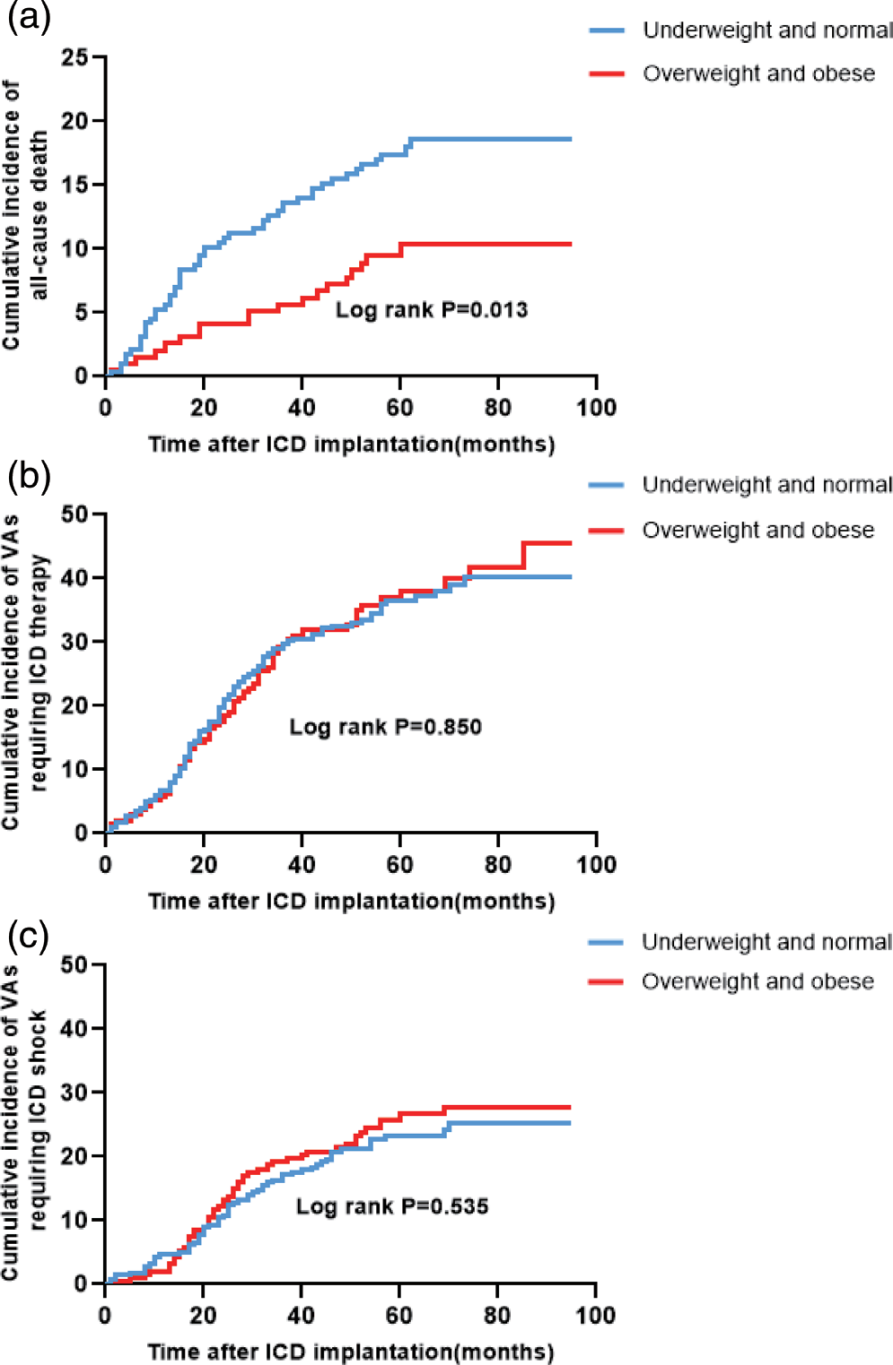
Kaplan‐Meier estimates of the cumulative incidence of A, all‐cause death; B, VAs requiring ICD therapy; and C, VAs requiring ICD shock. ICD, implantable cardioverter defibrillator; VAs, ventricular arrhythmias

The clinical outcomes of the patients in the two groups are presented in Table 2. During a median follow‐up of 61 (1‐95) (median, min‐max) months, 70 patients (14.6%) died, 173 patients (36%) experienced VAs requiring appropriate ICD therapy, and 112 patients (23.3%) were treated with ICD shock. The all‐cause death rate in the underweight and normal group was higher than that in the overweight and obese group (17.9% vs 9.7%, *P* = .013). The incidence of VAs requiring appropriate ICD therapy was equivalent between the two groups (underweight and normal group, 34.4% vs overweight and obese group, 38.5%; *P* = .316). In addition, 21.4% of patients in the underweight and normal group received appropriate ICD shock, and 26.2% of patients in the overweight and obese group received ICD shock therapies (*P* = .227). Kaplan‐Meier survival curves were plotted to determine survival probability, VAs among patients based on BMI (Figure 1). The cumulative incidence of all‐cause death was lower in the overweight and obese group (log‐rank *P* = .013), while the cumulative incidence of VAs requiring ICD therapy or shock in the overweight and obese group was not significantly different from that in the underweight and normal group (VAs requiring ICD therapy, log‐rank *P* = .850; VAs requiring ICD shock, log‐rank *P* = .535).

**TABLE 2:** Clinical outcomes of patients depending on BMI

	All	Underweight and normal (BMI < 24 kg/m^2^)	Overweight and obese (BMI≥24 kg/m^2^)	*P* value
All‐cause death	70 (14.6%)	51 (17.9%)	19 (9.7%)	.013
VAs requiring ICD therapy	173 (36%)	98 (34.4%)	75 (38.5%)	.316
VAs requiring ICD shock	112 (23.3%)	61 (21.4%)	51 (26.2%)	.227

Abbreviations: BMI, body mass index; ICD, implantable cardioverter defibrillator; VAs, ventricular arrhythmias.

Cox regression modeling (Table 3) demonstrated that as a categorical variable, BMI≥24 kg/m^2^ (overweight and obese) was associated with a decreased risk of all‐cause death (hazard ratio [HR] 0.52, 95% confidence interval [CI] 0.31‐0.88; *P* = .015) in an unadjusted model. BMI≥24 kg/m^2^ (overweight and obese) remained an independent protective factor against all‐cause death (HR 0.44; 95% CI 0.26‐0.77; *P* = .003) after adjustment in a multivariate model (adjusted for age, New York Heart Association (NYHA) III‐IV, hypertension, diabetes, left ventricular end‐systolic dimension (LVEDD), left ventricular ejection fraction (LVEF), loop diuretic use, and spironolactone use). However, the risk of VAs requiring appropriate ICD therapy or shock was similar in both groups in univariate and multivariate Cox models.

**TABLE 3:** Univariate and multivariate Cox proportional hazards regression analysis of clinical outcomes according to categorical BMI

	Univariate HR (95% CI)	*P* value	Multivariate HR (95% CI)	*P* value
All‐cause death				
Overweight and obese (BMI ≥24 kg/m^2^)	0.52 (0.31–0.88)	0.015	0.44 (0.26–0.77)	.003
VAs requiring ICD therapy				
Overweight and obese (BMI≥24 kg/m^2^)	1.05 (0.78‐1.42)	0.759	0.95 (0.69‐1.29)	.945
VAs requiring ICD shock				
Overweight and obese (BMI≥24 kg/m^2^)	1.17 (0.81‐1.70)	0.401	1.09 (0.74‐1.59)	.663

Abbreviations: BMI, body mass index; CI, confidence interval; HR, hazard ratio; ICD, implantable cardioverter defibrillator; VAs, ventricular arrhythmias.

### 3.3 Univariate and multivariate risk factors of all‐cause death and VAs

**FIGURE 2:**
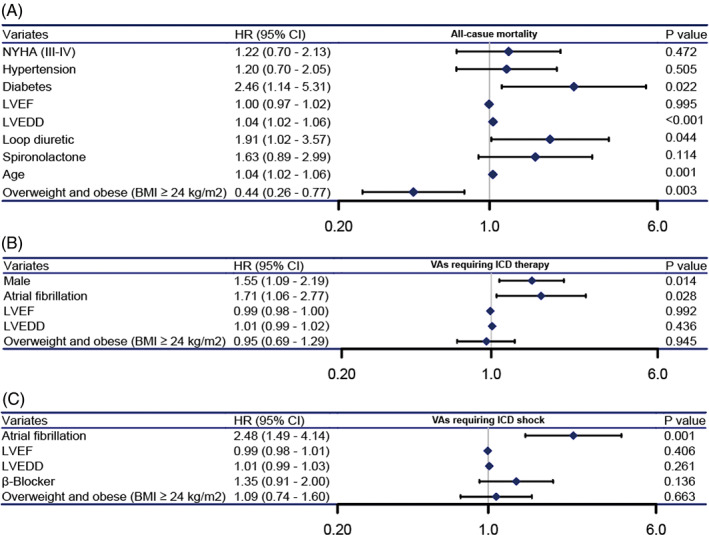
Forest plot illustrating the HR and 95% CI of A, all‐cause mortality; B, VAs requiring ICD therapy; and C, VAs requiring ICD shock using the multivariate Cox model. CI, confidence interval; HR, hazard ratio; ICD, implantable cardioverter defibrillator; VAs, ventricular arrhythmias; other abbreviations as in Table 1

Univariate Cox proportional hazards models of all‐cause death and VAs are shown in Supplementary Table S1. Older age, NYHA (III‐IV), hypertension, diabetes, lower LVEF, wider LVEDD, loop diuretic use, and spironolactone use were univariate predictors of all‐cause death in the overall group, while BMI ≥24 kg/m^2^ (overweight and obese) was a predictor of improved survival. Variables that were entered into the multivariate Cox model of all‐cause death are shown in Figure 2A. Diabetes (HR 2.46; 95% CI 1.14‐5.3; *P* = .022), LVEDD (HR 1.04; 95% CI 1.02‐1.06; *P* < .01), loop diuretic use (HR 1.91; 95% 1.02‐3.57; *P* = .044), and older age (HR 1.04; 95% CI 1.0‐1.06; *P* = .001) were independent predictors of increased mortality, while BMI ≥2 4 kg/m^2^ (overweight and obese) was an independent predictor of improved survival. Male, AF, lower LVEF, and wider LVEDD were univariate predictors of VAs requiring ICD therapy; moreover, AF, lower LVEF, wider LVEDD, and β‐blocker use were univariate predictors of VAs requiring ICD shock. The variables above and BMI ≥24 kg/m^2^ (overweight and obese) that were entered into the multivariate Cox model of VAs or shocks are shown in Figure 2B,C. Male (HR 1.55; 95% CI 1.09‐2.19; *P* = .014) and AF (HR 1.71; 95% CI 1.06‐2.77; *P* = .028) were independent predictor of VAs requiring ICD therapy; AF (HR 2.48; 95% CI 1.49‐4.14; *P* = .001) was an independent predictor of VAs requiring ICD shock.

## 4 DISCUSSION

The major findings of this study are as follows: (a) over the median follow‐up of 5 years, overweight and obese patients have higher survival than underweight and normal range patients; and (b) overweight and obesity have only a neutral impact on VAs risk in NICM patients with an ICD equipped with an HM system.

Our study demonstrates that the obesity paradox of survival exists in NICM patients with an ICD. This phenomenon is similar in patients diagnosed with diseases, such as AF,^9^ hypertrophic cardiomyopathy,^24^ heart failure,^6^, ^7^, ^10^, ^11^ hypertension,^8^ and CAD.^4^ Several studies have illustrated the impact of BMI on mortality in ICD patients, but the results are inconsistent.^12^, ^13^, ^14^, ^15^ Kenneth et al. reported that low BMI was independently associated with death within a year.^12^ In a retrospective analysis of patients diagnosed with left ventricular dysfunction after healing from myocardial infarction from the Multicenter Automatic Defibrillator Implantation Trial‐II (MADIT II), obese patients (BMI≥30 kg/m^2^) had better survival than nonobese patients.^13^ Another study from the United States found that a higher BMI produced a higher survival benefit in ICD patients, especially elderly patients, during a mean follow‐up of 2.6 years.^14^ Our study had a median follow‐up of 5 years, suggesting that the beneficial impact of BMI could extend to such a long period. In addition, our study also found that older age was an independent risk factor for mortality, which was consistent with the results of the previous study.^14^ However, a multicenter retrospective study conducted in Spanish hospitals found that BMI was not associated with mortality in patients with a primary prevention ICD.^15^ The reason for the inconsistency of the results may be mainly due to the differences in the population characteristics, racial groups, sample size, and covariates for adjustment. Our study enrolled only Chinese NICM patients, who were quite different from patients in previous studies. Furthermore, in our study, we found that patients with diabetes had an almost 1.5‐fold increased risk of mortality compared with those without diabetes. In the study evaluating death within a year in ICD patients, diabetes was an independent risk factor for mortality.^12^ Therefore, our study extended Kenneth et al^12^ findings to a follow‐up of 5 years, suggesting that the negative effect of diabetes on survival in ICD patients lasted for a long period. This reminded us of the importance of preventing and managing diabetes in ICD patients.

In our study, all ICDs were equipped with an HM system, which allowed us to easily and precisely record the VAs. Through the data from the HM system, we found that BMI was not associated with the risk of VAs in NICM patients with an ICD. Only a few studies have investigated the impact of BMI on VAs in patients with ICD. Pietrasik et al performed a post hoc analysis of nondiabetic patients with ischemic left ventricular dysfunction from the MADIT II and found that obese patients (BMI≥30 kg/m^2^) suffered a higher rate of VAs compared with nonobese patients.^16^ However, retrospective analysis of subgroup ICD patients from the multicenter automatic defibrillator implantation trial with cardiac resynchronization therapy (MADIT‐CRT) suggested that BMI had no influence on the VAs in ICD patients.^17^ The contradictory results of these two studies may be due to different study populations. The MADIT II study enrolled only ischemic cardiomyopathy (ICM) patients, while the MADIT‐CRT study enrolled patients with both ICM and NICM. Our study included only NICM patients with an ICD, and the neutral effect of BMI was detected on VAs. Moreover, in our study, we found that the presence of AF was independently associated with higher VAs, which suggested that we should pay special attention to AF in NICM patients with an ICD.

The explanation for the obesity paradox of mortality is still unclear in ICD patients. In our study, we found that higher BMI has a protective effect against mortality but not against VAs. Our results indirectly suggest that the protective effect of BMI against death is not achieved by reducing VAs. For patients with advanced cardiovascular diseases, some studies have proposed some viewpoints about the mechanism behind the obesity paradox.^3^, ^7^, ^11^ In patients with heart failure, a higher BMI might be a protector against cachexia, which promotes mortality.^25^ A higher BMI means a better energy reserve and nutrition level,^26^ which is associated with a beneficial prognosis.^27^ Therefore, the inverse relationship between obesity and mortality in NICM patients with an ICD may stem from the better nutritional status of obesity.

Our study has several limitations. First, this was a multicenter, retrospective, observational study that was subject to bias. However, we used a multivariable Cox model to minimize the bias. Second, our study focused on NICM, resulting in a relatively small number of samples. Due to the low proportion of underweight and obese patients, we did not divide the whole patient population into four groups based on BMI for analysis. If the sample size had been sufficient, we could have had a better evaluation of the effect of each group on clinical outcomes. Third, we did not collect information on adiposity distribution (waist‐to‐hip ratio or waist circumference) or percentage of body fat in our study. It would be meaningful to compare the effects of peripheral obesity and abdominal obesity on clinical outcomes. However, previous studies have shown that the obesity paradox of mortality still exists, regardless of the measures of adiposity.^28^, ^29^ Finally, we had information on BMI before ICD implantation only, and the change in BMI during the follow‐up was unknown. And previous study reported that higher body weight fluctuations were associated with adverse outcomes in CAD patients.^30^ Thus, a prospective study is needed to explore the dynamic influence of BMI on clinical outcomes in NICM patients with an ICD.

## 5 CONCLUSION

Overweight and obesity are protective against all‐cause death but have only a neutral impact on VAs compared with normal weight and underweight in NICM patients with an ICD. This information is significantly meaningful. The conclusions from our study suggest that a higher BMI may be beneficial to survival and does not increase VAs risk.

## CONFLICT OF INTEREST

The authors declare no potential conflict of interests.

## Supporting information


**Supplementary Table 1** Univariate Cox proportional hazards regression analysis of clinical outcomes

## Data Availability

The data that support the findings of this study are available from the corresponding author upon reasonable request.
