# Supplementary material for: Overweight and obesity as protective factors against mortality in nonischemic cardiomyopathy patients with an implantable cardioverter defibrillator
Source: Clin Cardiol. 2020 Sep 16;43(12):1435–42. doi: 10.1002/clc.23458 (PMC7724223; doi:10.1002/clc.23458)
Supplement: Supplementary file 1 — Supplementary Table 1 Univariate Cox proportional hazards regression analysis of clinical outcomes [file CLC-43-1435-s001.docx]

**Supplementary Table 1 Univariate Cox proportional hazards regression analysis of clinical outcomes**

| Variables | **All-cause death** | | VAs requiring ICD therapy | | VAs requiring ICD shock | | |
| --- | --- | --- | --- | --- | --- | --- | --- |
|  | HR (95% CI) | P | HR (95%CI) | P | HR (95% CI) | | P |
| Overweight and obese (BMI≥24 kg/m^2^) | 0.52(0.31-0.88) | 0.015 | 1.05(0.78-1.42) | 0.759 | | 1.17(0.81-1.70) | 0.401 |
| Age, years | 1.04(1.02-1.06) | ＜0.001 | 0.99(0.98-1.00) | 0.093 | | 0.99(0.98-1.00） | 0.051 |
| Male | 1.41(0.83-2.41） | 0.209 | 1.52(1.08-2.13） | 0.017 | | 1.32(0.88-2.00） | 0.184 |
| NYHA (III-IV) | 2.69(1.68-4.31） | ＜0.001 | 1.20(0.88-1.65） | 0.249 | | 1.23(0.83-1.82） | 0.294 |
| Primary prevention | 1.57(0.98-2.53） | 0.063 | 0.94(0.70-1.28） | 0.707 | | 1.05(0.72-1.53） | 0.784 |
| QRS duration, ms | 1.01(1.00-1.02） | 0.076 | 1.00(0.99-1.00） | 0.457 | | 1.00(0.99-1.01） | 0.797 |
| Hypertension | 1.80(1.09-2.99） | 0.022 | 0.79(0.53-1.17） | 0.233 | | 0.95(0.60-1.52） | 0.844 |
| Diabetes | 2.57(1.23-5.37） | 0.012 | 1.00(0.49-2.03) | 0.998 | | 1.12(0.49-2.54） | 0.794 |
| Atrial fibrillation | 1.28(0.59-2.79） | 0.537 | 1.84(1.15-2.94） | 0.010 | | 2.91(1.78-4.77） | ＜0.001 |
| Stoke | 0.05(0.000-948.883） | 0.549 | 1.20(0.30-4.85） | 0.795 | | 1.74(0.43-7.06） | 0.436 |
| Preimplant syncope | 1.09(0.64-1.88) | 0.732 | 1.35(0.97-1.87） | 0.077 | | 1.29(0.85-1.94） | 0.227 |
| LVEF, % | 0.97(0.95-0.98) | ＜0.001 | 0.99(0.98-0.99） | 0.004 | | 0.98(0.97-1.00） | 0.011 |
| LVEDD, mm | 1.05(1.03-1.06） | ＜0.001 | 1.02(1.00-1.03） | 0.017 | | 1.02(1.00-1.04） | 0.012 |
| β-Blocker | 1.44(0.89-2.34） | 0.140 | 1.35(1.00-1.83） | 0.053 | | 1.50(1.02-2.20） | 0.039 |
| Amiodarone | 0.96(0.57-1.60） | 0.861 | 1.15(0.83-1.58） | 0.398 | | 1.25(0.84-1.84) | 0.272 |
| ACEI or ARB | 1.50(0.91-2.45) | 0.109 | 0.87(0.69-1.37） | 0.867 | | 1.25(0.83-1.87) | 0.289 |
| Loop diuretic | 3.39(2.09-5.48) | ＜0.001 | 1.03(0.69-1.56） | 0.878 | | 1.31(0.82-2.11) | 0.262 |
| Spironolactone | 2.98(1.86-4.78） | ＜0.001 | 1.24(0.88-1.76） | 0.225 | | 1.51(1.00-2.28) | 0.051 |

Abbreviations: ACEI, angiotensin-converting enzyme inhibitor; ARB, angiotensin receptor blocker; BMI, body mass index; CI, confidence interval; HR, hazard ratio; ICD, implantable cardioverter deﬁbrillator; LVEF, left ventricular ejection fraction; LVEDD, left ventricular end-systolic dimension; NYHA, New York Heart Association; VAs, ventricular arrhythmias
